# Supplementary material for: Structural Characterization and Assessment of Anti-Inflammatory Activities of Polyphenols and Depsidone Derivatives from Melastoma malabathricum subsp. normale
Source: Molecules. 2022 Feb 24;27(5):1521. doi: 10.3390/molecules27051521 (PMC8912040; doi:10.3390/molecules27051521)
Supplement: Supplementary file 1 [file molecules-27-01521-s001.zip › molecules-1597952-supplementary.pdf]

Supplementary data

Structural characterization and assessment of anti-inflammatory activities of polyphenols and depsidone derivatives from *Melastoma malabathricum subsp. normale*

Rui-Jie He [1], Ya-Feng Wang [1], Bing-Yuan Yang [1], Zhang-Bin Liu [1], Dian-Peng Li [1], Bi-Qun Zou[2,]*, Yong-Lin Huang [1,]*

*Correspondence: zoubiqun@163.com (B.-Q. Zou); hyl@gxib.cn(Y.-L.H.); Tel.: +86-773-355-0829; Fax: +86-773-355-0067

Figure S1. ^{1}H NMR (500 MHz, Acetone-d_6) spectrum of **1**
Figure S2. ^{13}C NMR (125 MHz, Acetone-d_{64}) spectrum of **1**
Figure S3. HSQC spectrum of **1** in Acetone-d_6
Figure S4. HMBC spectrum of **1** in Acetone-d_6
Figure S5. ^{1}H-^{1}H COSY spectrum of **1** in Acetone-d_6
Figure S6. Roesy spectrum of **1** in Acetone-d_6
Figure S7. HR-ESI-MS spectrum of **1**
Figure S8. CD spectrum of **1**
Figure S9. The ^{1}H NMR spectrum of **11** (500 MHz, DMSO-d_6)
Figure S10. The ^{13}C NMR spectrum of **11** (125 MHz, DMSO-d_6)
Figure S11. The HSQC spectrum of **11** (500 MHz, DMSO-d_6)
Figure S12. The HMBC spectrum of **11** (500 MHz, DMSO-d_6)
Figure S13. The HR-ESI-MS spectrum of **11**

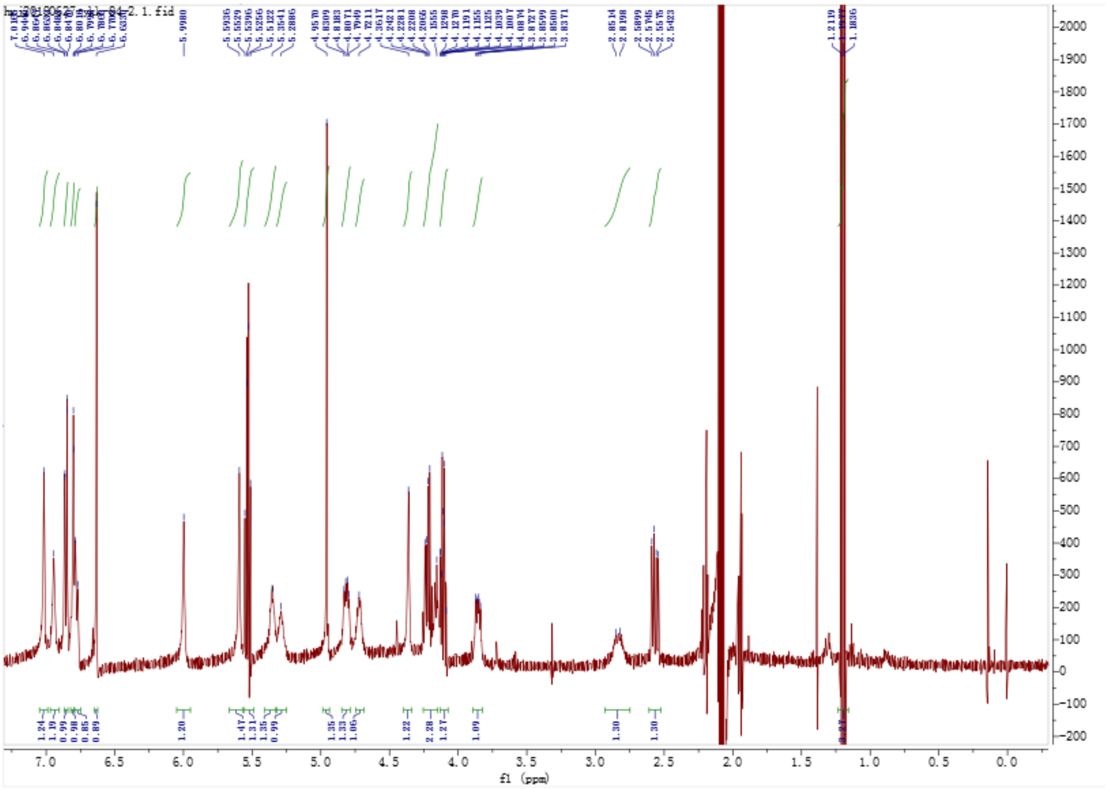

Figure S1. ^{1}H NMR (500 MHz, Acetone-d_6) spectrum of **1**

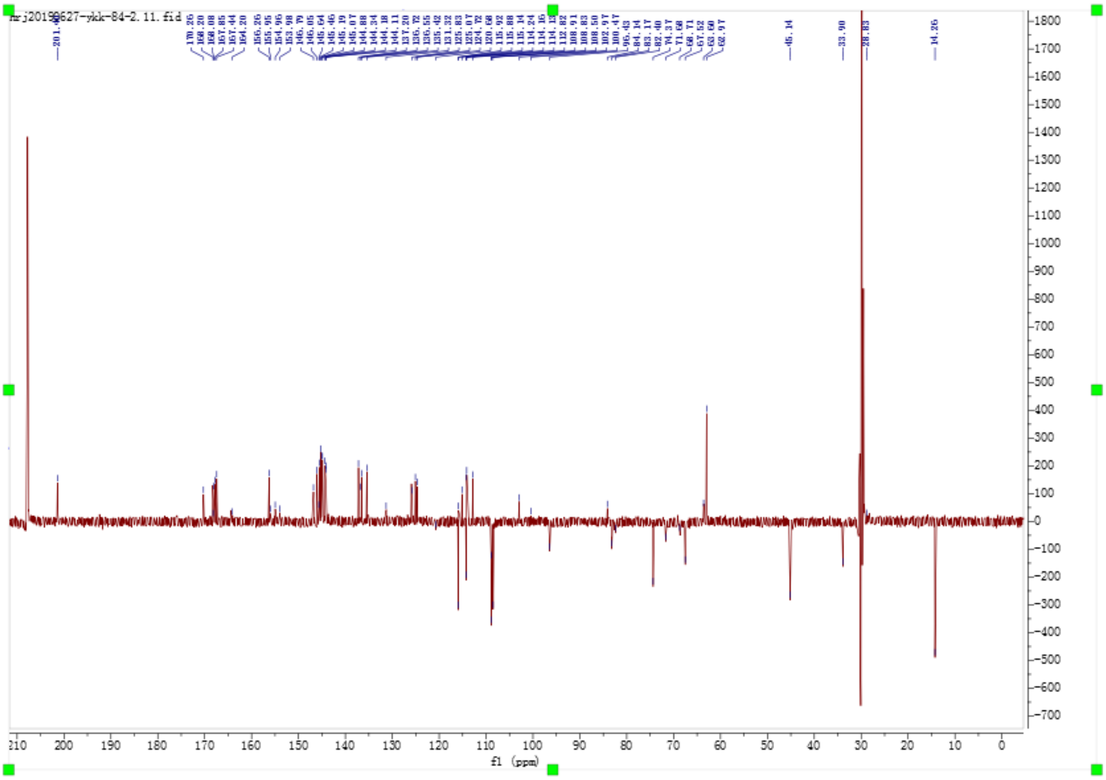

Figure S2. ^{13}C NMR (125 MHz, Acetone-d_6) spectrum of **1**

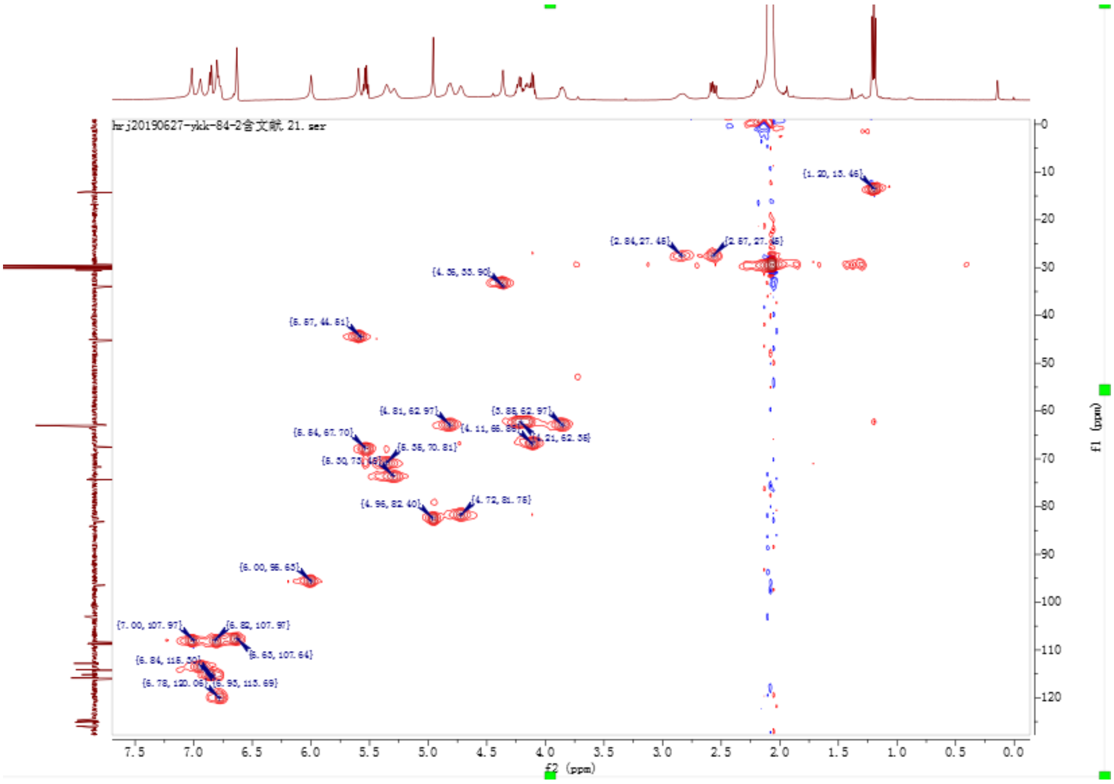

Figure S3. HSQC spectrum of **1** in Acetone-d_6

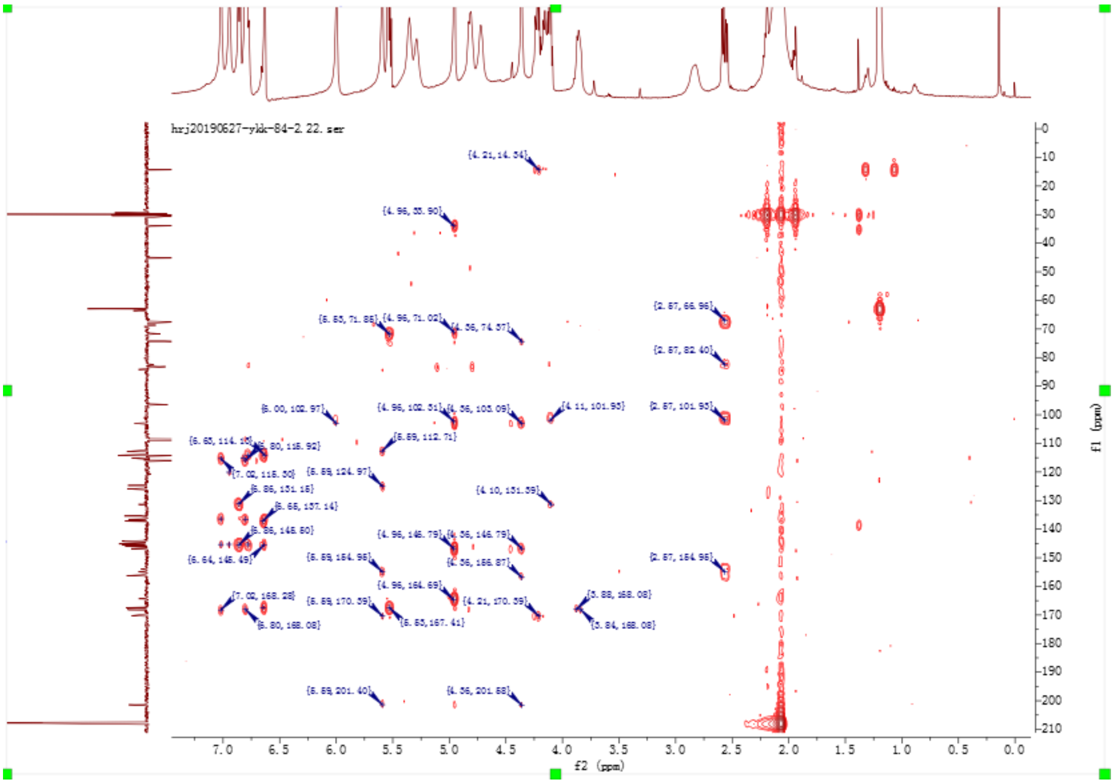

Figure S4. HMBC spectrum of **1** in Acetone-d_6

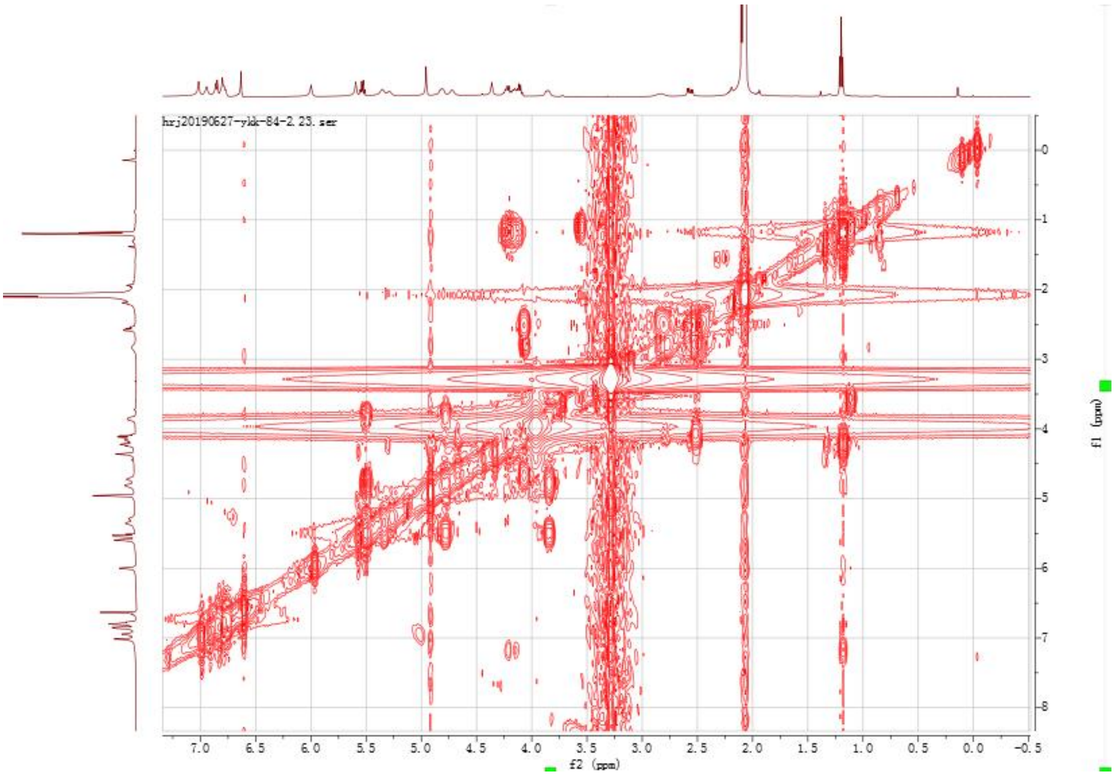

Figure S5. ^{1}H-^{1}H COSY spectrum of **1** in Acetone-d_6

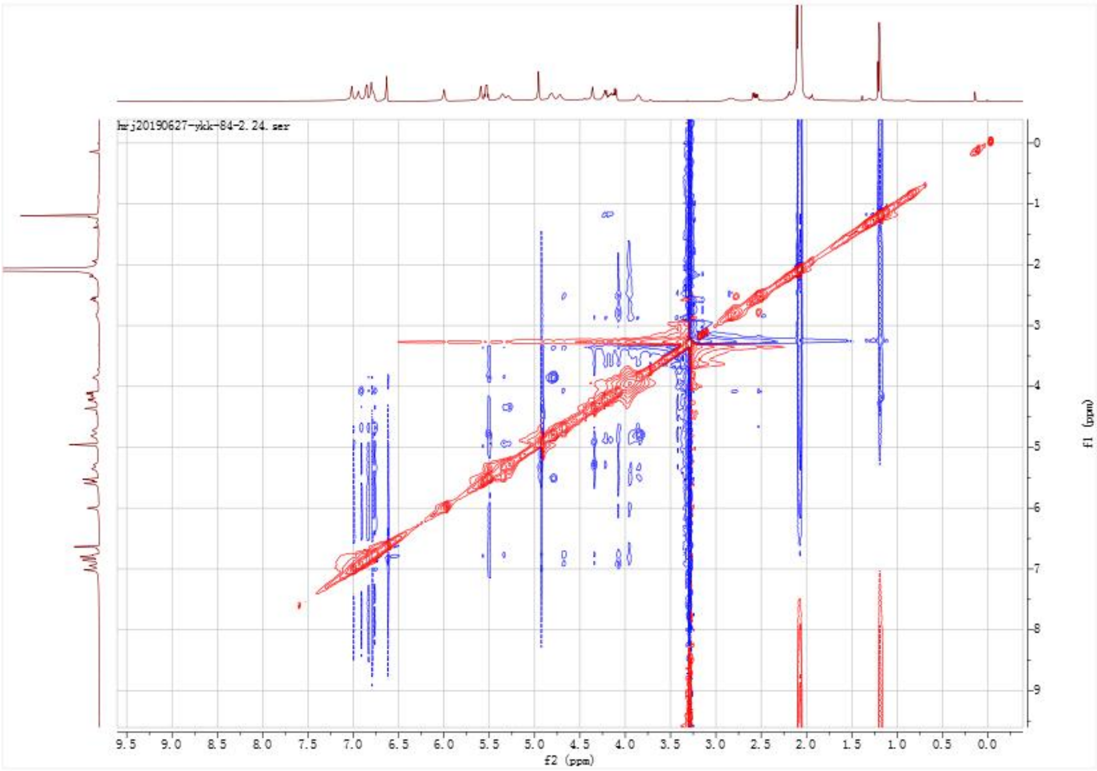

Figure S6. Roesy spectrum of **1** in Acetone-d_6

Elmt	Val.	Min	Max
H	1	1	100
C	4	1	100
N	3	0	0

Elmt	Val.	Min	Max
O	2	0	100

Use Adduct
H

Error Margin (ppm): 10
HC Ratio: 0.1 - 4.0
Max Isotopes: all
MSn Iso RI (%): 75.00

DBE Range: 1.0 - 50.0
Apply N Rule: yes
Isotope RI (%): 1.00
MSn Logic Mode: AND

Electron Ions: both
Use MSn Info: no
Isotope Res: 10000
Max Results: 500

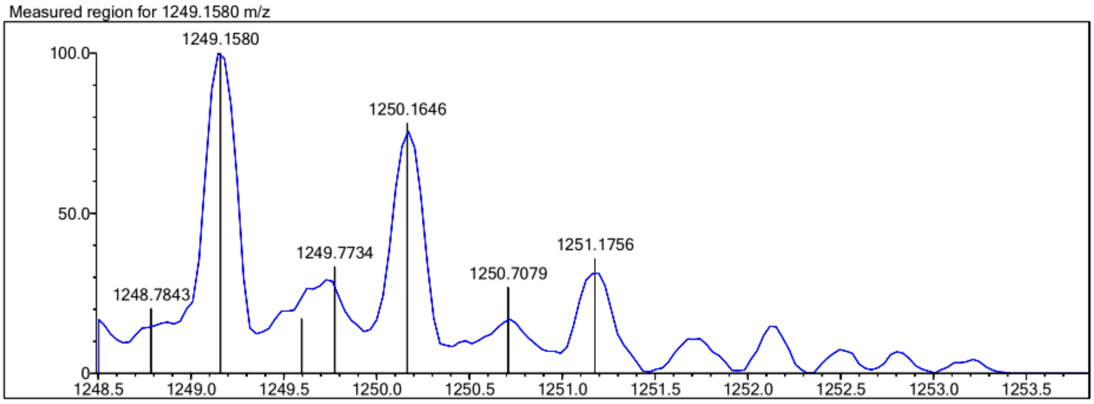

Rank	Score	Formula (M)	Ion	Meas. m/z	Pred. m/z	DBE	Df. (ppm)	Iso	Meas. (M)
2	42.26	C58 H42 O32	[M-H]-	1249.1580	1249.1586	38.0	-0.48	42.26	1250.1653

Figure S7. HR-ESI-MS spectrum of **1**

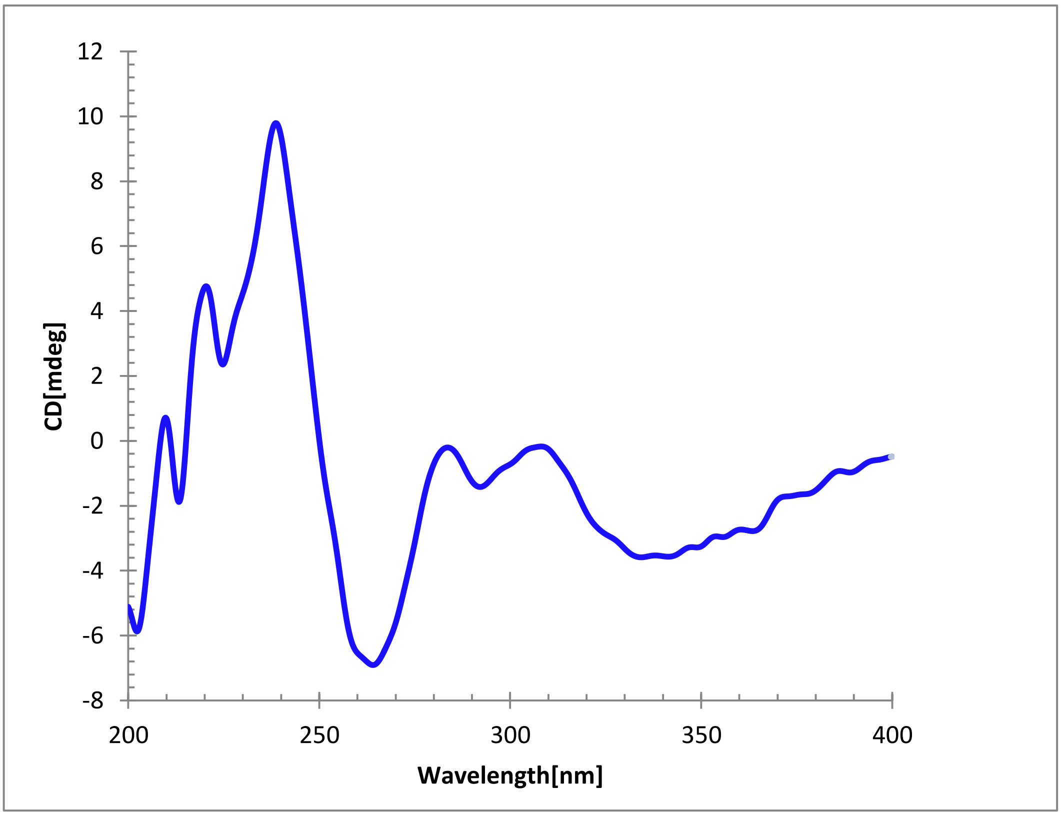

Figure S8. CD spectrum of **1**

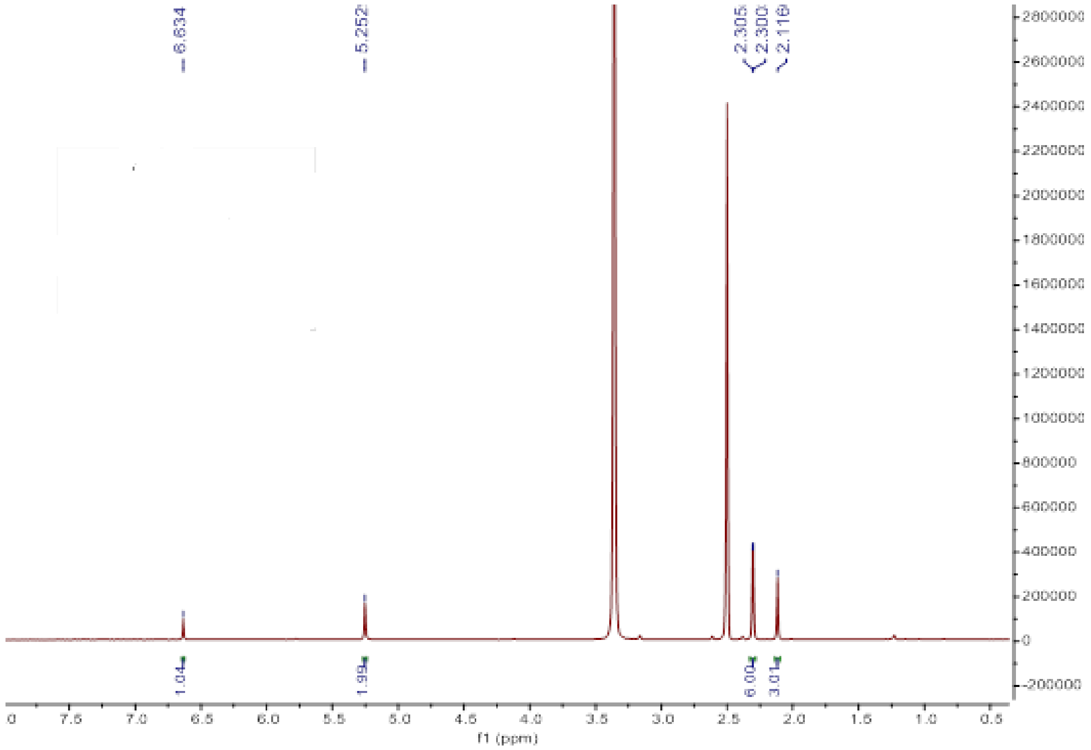

Figure S9 The ^{1}H NMR spectrum of **11** (500 MHz, DMSO-d_6)

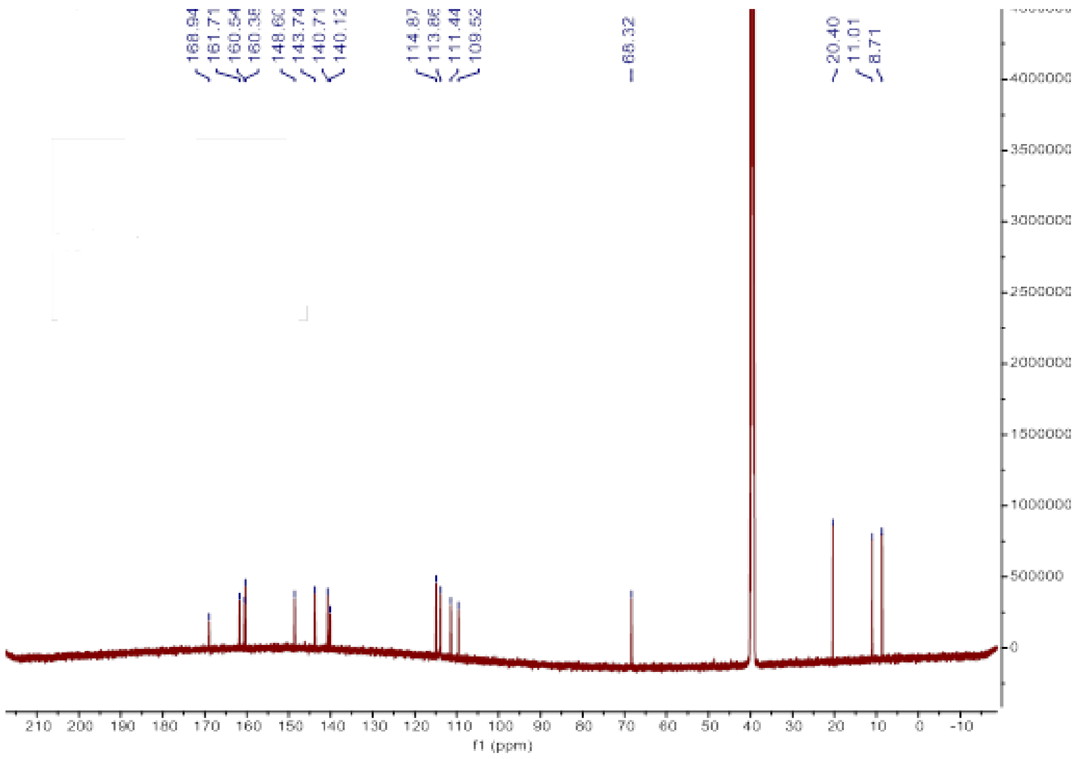

Figure S10 The ^{13}C NMR spectrum of **11** (125 MHz, DMSO-d_6)

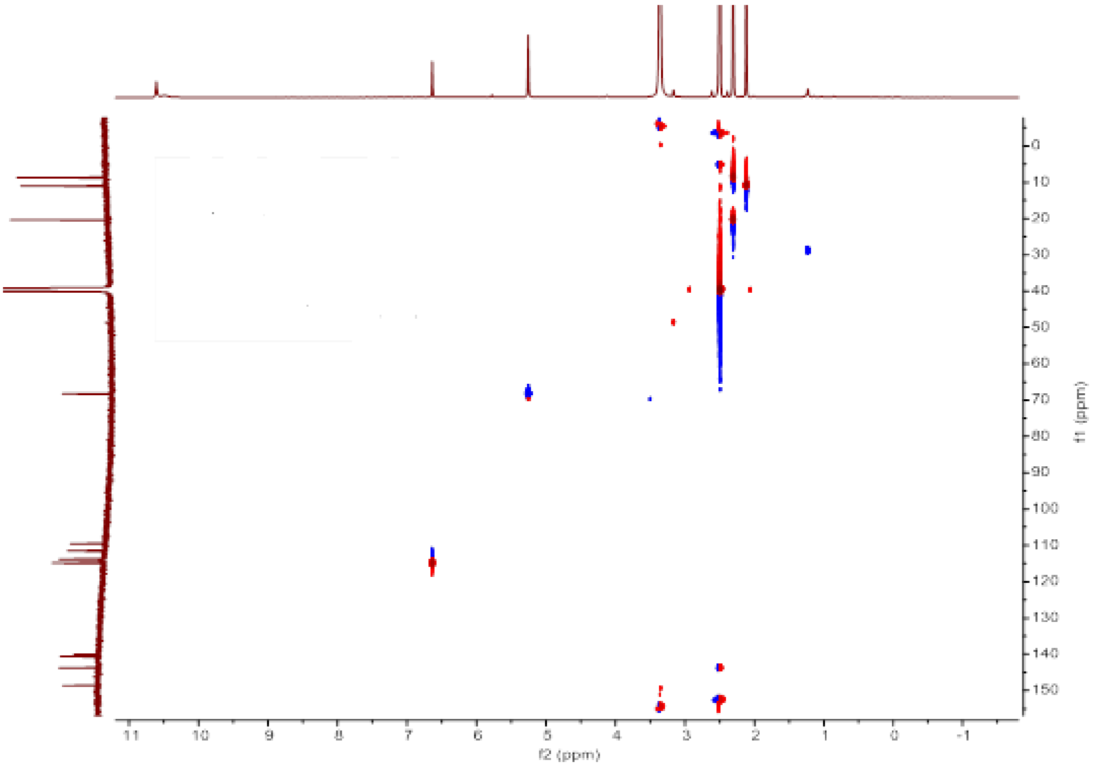

Figure S11 The HSQC spectrum of **11** (500 MHz, DMSO-d_6)

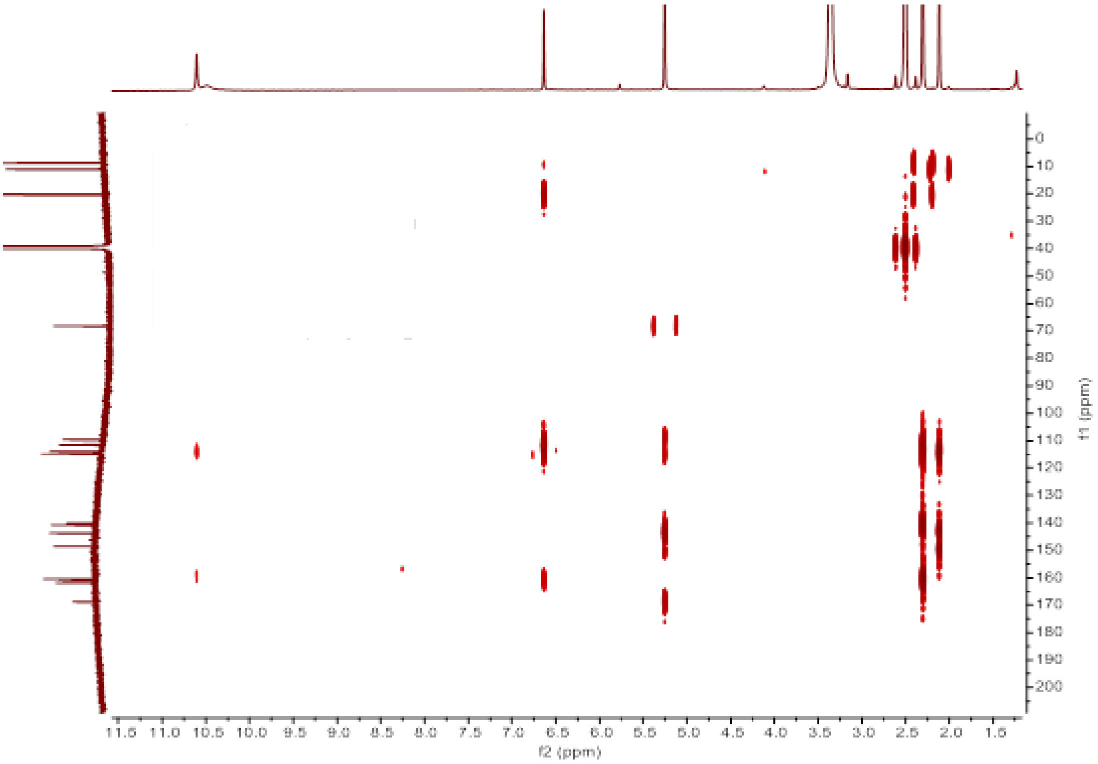

Figure S12 The HMBC spectrum of **11** (500 MHz, DMSO-d_6)

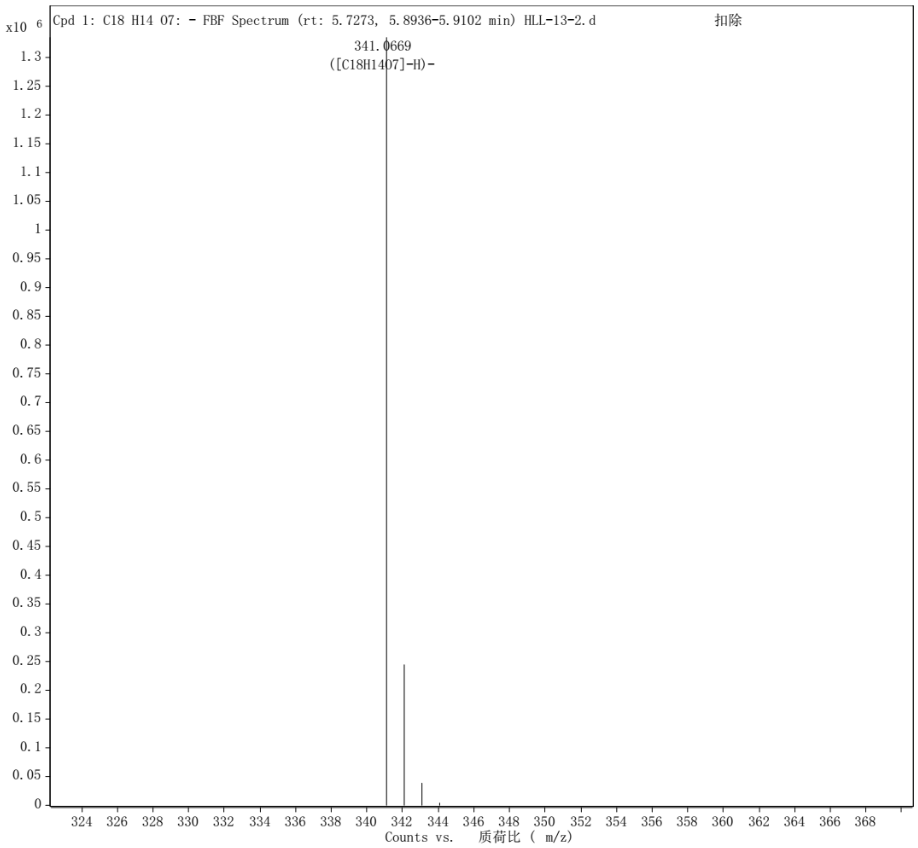

Figure S13 The HR-ESI-MS spectrum of **11**
